# Management of aromatase inhibitor–induced arthralgia

**DOI:** 10.3747/co.v17i1.474

**Published:** 2010-02

**Authors:** J. Younus, L. Kligman

**Keywords:** Aromatase inhibitors, arthritis, arthralgia, breast cancer

## Abstract

Aromatase inhibitors (ais) are commonly used as adjuvant treatment in postmenopausal women with hormone receptor–positive early breast cancer. With both steroidal and nonsteroidal ais, ai-induced arthralgia is frequently observed. The mechanism of ai-induced arthralgia remains unknown, and the data available from clinical trails using ais are limited. We review the pertinent information from a clinical perspective, including an algorithm to treat ai-induced arthralgia.

## 1. INTRODUCTION

Arthralgia is a symptom commonly seen in patients treated with any aromatase inhibitor (ai). Trials with ais have used various terms to describe joint discomfort. Table I summarizes the incidence of that symptom in various clinical trials.

## 2. RISK FACTORS

Although no known risk factors (see Table II) uniformly emerged in the trials with ais, patients treated with adjuvant chemotherapy in the atac (Arimidex Alone or in Combination with Tamoxifen) trial had a somewhat higher incidence and earlier onset of arthralgia 6,7. Increasing use of granulocyte colony–stimulating factor (g-csf) and taxane chemotherapies 8 in the adjuvant setting are seen as additional risk factors. In the atac trial, previous arthritis or arthralgia symptoms were seen in 46% of patients who developed arthralgia after initiation of anastrozole therapy. Among those patients, a relatively younger age group had a higher incidence of joint symptoms 6,7.

## 3. CLINICAL DESCRIPTION

Patients usually present with morning stiffness and joint discomfort in various sites, including hands, knees, back, hips, and shoulders. The clinical examination should pay particular attention to the affected joints and periarticular structures. Any signs of inflammation—such as tenderness, warmth, swelling, or erythema—should be noted. In our experience, no particularly consistent abnormality affiliated with the use of ais is observed on joint examination. No tests specifically address the diagnosis of ai-induced arthralgia. Plain radiographs and other related tests can be used to rule out other causes of arthralgia. The differential diagnosis should include bone disease, osteoarthritis, rheumatoid arthritis, and periarticular pathologies, among other possibilities.

Arthralgia has been observed to begin appearing at approximately 2 months after the start of treatment and to peak at around the 6-month mark, but it can appear up to 2 years after initiation of therapy 6. In the atac trial, the intensity of discomfort was rated between mild and moderate in 92% of patients 6. Users of anastrozole and tamoxifen both experienced a similar incidence (about 10%) of disabling events related to such joint symptoms. In atac, the withdrawal rate because of arthralgia was quoted as only 2.1% in patients treated with anastrozole, but other investigators have reported a higher incidence. In the report by Presant *et al.* 9, 20% of patients discontinued an ai because of the severity of arthralgia. On average, their pain intensity was rated as 7.5 out of 10. Crew *et al.* 10 reported that, of 200 patients treated with ais, 47% reported joint symptoms. In that survey, patients who had a higher body mass index (25–30 m^2^/kg^2^) and those who had been previously treated with tamoxifen were less likely to experience joint symptoms when treated with an ai. Patients previously treated with taxanes were four times more likely to develop arthralgia.

The resolution of arthralgias in various trials appears to begin around the 6-month mark from the onset of therapy, improving in at least 50% of patients 6. By 18 months, 75% of the patients in the atac trial had experienced significant amelioration of their symptoms 6,7.

## 4. POTENTIAL MECHANISMS BEHIND AI-INDUCED ARTHRALGIA

The acute drop in estrogen produced by aromatase inhibition is the most likely underlying mechanism for joint symptoms. That hypothesis is supported by the observation that backaches, joint pains, and stiffness are commonly experienced among peri- and postmenopausal women in the general population 8. The development of arthralgia after chemotherapy, which also causes hormone suppression, has been well documented 10,11.

No structural damage to articular surfaces, ligaments, or other joint structures has been documented with ai-induced arthralgia (unlike the case with osteoarthritis or rheumatoid arthritis). In a small prospective study, 12 patients on ais were followed by questionnaire, examination, and magnetic resonance imaging of hands and wrists at baseline and after 6 months. No patient had joint symptoms before treatment. After 6 months, these patients showed enhancement of the synovium and an increase in intra-articular and tendon sheath fluid, with associated complaints of stiffness in the small joints of the wrists and hands 12. That finding correlates well with the relative absence of signs of arthritis in patients with ai-induced arthralgia.

## 5. TREATMENT OPTIONS AND ALGORITHM

**FIGURE 1:**
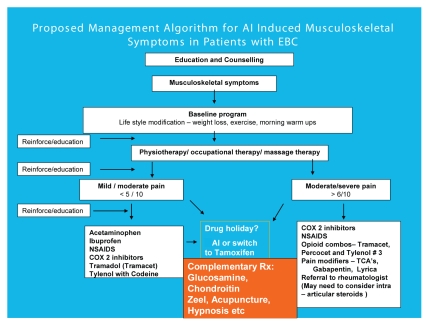
Proposed management algorithm for aromatase inhibitor (ai)–induced musculoskeletal symptoms in patients with early breast cancer (ebc). nsaids = nonsteroidal anti-inflammatory drugs; COX2 = cyclooxygenase 2; Δ = change; Rx = prescription; tcas = tricyclic antidepressants.

Aromatase inhibitor–induced arthralgia is a relatively newly recognized entity. It is reasonable to extrapolate and draw data from the osteoarthritis and rheumatoid arthritis literature for the overall management of joint symptoms seen in patients on an ai. But because the exact mechanism of the symptoms is unknown, these borrowed strategies may or may not work. Therefore the algorithm presented here (Figure 1) contains a combination of an evidence-based clinical approach and our own clinical experience.

Counselling and education appear to be the two most important steps in the overall management of these patients. It should be emphasized that therapy with an ai is a long-term plan, usually 5 years. Moreover, it has been observed as an emerging trend that many treating physicians may elect to continue ais for an even longer duration, depending on the risk of recurrence at initial presentation and other factors. The side effects should therefore be described clearly from beginning. Follow-up should be scheduled at 2 months and at 6 months from the date of ai therapy initiation. Further follow-up and counselling may depend on the individual patient’s symptoms. The clinician should emphasize the clear benefit of reducing the risk of breast cancer recurrence and the importance of adherence to the long-term use of an ai at each patient visit. Several available options to treat ai-induced arthralgia should be discussed with patients, depending on the severity of symptoms.

For patients with ai-induced arthralgia, simple lifestyle modifications such as weight reduction and regular exercise, with increasing joint mobility, may help them to regain muscle strength and may improve fatigue, posture, and flexibility. Improvement in function and pain scores in patients with osteoarthritis of the knee has been shown in research trials of weight loss and exercise, with the combination of the two interventions being more effective than either one alone. A 10% weight reduction in such patients improved function by almost 30% 13,14. Similar benefits were reported in trials of hydrotherapy 15,16.

Massage therapy using the Swedish technique has been demonstrated to reduce pain and improve function for patients with symptomatic osteoarthritis of the knee 17. Relaxation techniques, biofeedback, and visual imagery can also be helpful 18. A pilot study of the effectiveness of guided imagery combined with progressive muscle relaxation has shown promise as an effective self-management technique for chronic pain associated with osteoarthritis 19.

Among non-pharmaceutical interventions, hypnosis remains an under-investigated but potentially effective therapy. In clinical practice, a combination of hypnosis with other strategies may prove more effective, particularly in refractory cases. A case report on arthralgia and myalgias recently published by our group demonstrated the positive effect of adding hypnosis 20.

No trials of the foregoing modifications have involved patients with ai-induced arthralgia, but it may be reasonable to suggest them, based on similar symptoms felt by the patients who were studied and the improvement observed. Most of the adjuvant ai trials incorporated the use of calcium and vitamin D supplements. Severe hypovitaminosis D has been reported to be prevalent in patients with persistent nonspecific musculoskeletal pain and has been postulated as a potential cause of pain in women on ais 21. The use of these two supplements in patients with low levels of vitamin D has been shown to improve musculoskeletal symptoms 22.

To control the discomfort, acetaminophen alone should be tried first. Ibuprofen or other nonsteroidal anti-inflammatory drugs can be added for continued pain. The addition of opioids with or without acetaminophen can be used for more severe or nonresponsive pain. Pain modifiers such as tricyclic antidepressants or anticonvulsants may be added for severe, resistant, painful arthralgia. For complex situations or puzzling diagnostic issues, a rheumatology or pain management consultation is appropriate.

Occasionally, it is difficult to be clinically confident that the arthralgia is associated with ai use. A drug holiday for 2–3 weeks may safely be suggested to help resolve this issue.

Many complementary therapies have been tried in various arthritic conditions with mixed results. Acupuncture and homeopathic preparations have both shown some promise in alleviating arthritis pain 23,24. In a meta-analysis that considered more than 700 patients, chondroitin sulphate used in double-blind fashion for 2–12 months at doses of 800–2000 mg daily was shown to improve osteoarthritic pain. An interesting study report by Bruyere *et al.* 25 randomized 319 postmenopausal women to glucosamine or placebo and followed them prospectively for 3 years. The total knee replacement rate was almost 2.5 times higher in the placebo-treated group. Furthermore, joint space narrowing, quality of life, pain, and function related to the joints were better with glucosamine-supplemented participants.

Recently, two studies examined improvement in joint symptoms after patients were switched from one nonsteroidal ai to another. Close to 180 patients who were receiving an adjuvant ai and were complaining of joint symptoms were switched to another ai (anastrozole to letrozole, or vice-versa) after 3 months of therapy 26. About half these patients experienced improvement or resolution of their symptoms after the switch. Almost 75% of patients who were switched to tamoxifen instead of another ai obtained relief. In the second report 27, 179 patients treated with adjuvant anastrozole who had experienced severe joint pain were switched to letrozole after a 1-month washout period. Two thirds of those patients were able to continue with letrozole treatment with improved symptoms. The results of the foregoing studies suggest that switching to another ai may be reasonable in difficult and resistant cases. If the arthralgia remains unresolved, a final switch to tamoxifen could be considered.

## 6. SUMMARY

Arthralgia associated with the use of ais is a complicated and challenging clinical problem for health care personnel. This side effect is common and threatens to adversely affect compliance unless managed appropriately. The algorithm presented here offers a reasonably comprehensive approach to managing this common side effect and attempts to keep patients compliant with their potentially life-saving therapy.

## Figures and Tables

**TABLE I:** Incidence of arthralgia in aromatase inhibitor trials

Reference	Trial short name	Arthralgia incidence (%) with			
		Anastrozole	Tamoxifen	Letrozole	Exemestane
Fallowfield *et al.,* 2004^1^	atac	35.9	29.8		
Thürlimann *et al.,* 2005^2^	big 1–98		13.5	20	
Coombes *et al.,* 2004^3^	ies		11.8		18.6
Jakesz *et al.,* 2005^4^	arno 95	16.8	8		
Whelan *et al.,* 2005^5^	ma.17	25	21		

**Table II:** Risk factors for arthralgia

Younger age
Adjuvant chemotherapy (use of taxanes in particular)
Use of granulocyte colony–stimulating factor
Prior history of arthralgia, arthritis, or fibromyalgia
